# Liquid biopsy in organ damage: introduction of an innovative tool for assessing the complexity of blunt splenic injury

**DOI:** 10.1186/s40001-024-02254-z

**Published:** 2025-01-04

**Authors:** Aliona Wöhler, Bingduo Wang, Robert Schwab, Veronika Lukacs-Kornek, Arnulf G. Willms, Miroslaw T. Kornek

**Affiliations:** 1 Department of General, Visceral and Thoracic Surgery, German Armed Forces Central Hospital, Koblenz, Germany; 2 Department of Internal Medicine I, University Hospital of the Rheinische Friedrich Wilhelms University, Venusberg-Campus 1, 53127 Bonn, Germany; 3 Institute of Molecular Medicine and Experimental Immunology, University Hospital of the Rheinische Friedrich Wilhelms University, Bonn, Germany

**Keywords:** Biomarker, Extracellular vesicles, Exosomes, NOM, Spleen laceration, Liquid biopsy, AAST, Blunt splenic injury, Trauma

## Abstract

Liquid biomarkers are essential in trauma cases and critical care and offer valuable insights into the extent of injury, prognostic predictions, and treatment guidance. They can help assess the severity of organ damage (OD), assist in treatment decisions and forecast patient outcomes. Notably, small extracellular vesicles, particularly those involved in splenic trauma, have been overlooked. Here, we reanalyzed our data and explored whether monocyte-derived small EVs are correlated with AAST score (American Association for the Surgery of Trauma) scoring and are sensitive enough to distinguish the severity of splenic trauma in our explorative study. There was a correlation between monocyte-derived small extracellular vesicles (EVs) and the AAST score (r_Sp_ = 0.82, *p* < 0.001). Specifically, we observed that blood-borne small EVs originating from monocytes (CD9^+^CD14^+^ sEVs) were directly correlated with AAST scores. These EVs were found to be significantly elevated in patients with complex spleen injuries (AAST I–IV with hemodynamic instability and need for operative management) in an incremental manner; these patients were typically classified as AAST grade II or higher, which frequently included accompanying solid organ injuries. Our initial discovery shows great promise and warrants further exploration. This could pave a novel future path for a new critical care management approach for splenic injuries. There may also be synergistic effects when combined with extended focused assessment with sonography in trauma (E-FAST) sonography, particularly in triage scenarios, where resource constraints prevent immediate access to a CT scan.

## Introduction

The spleen is the most commonly injured solid organ in polytrauma patients. The treatment strategy depends on the hemodynamic stability of the patient, associated injuries and severity of the splenic trauma [1]. In recent years, there has been a remarkable shift toward a conservative approach in the nonoperative management of blunt liver and spleen trauma. However, in contrast with other blunt injuries, splenic trauma can be fatal due to delayed ruptures [1]. Recently, we provided the first evidence that a specific subset of small extracellular vesicles derived from monocytes, distinguished by their immobilization of CD9 on a microchip and positive expression of CD14, was linked to polytrauma patients with an injury severity score (ISS) greater than 15 and who had leading blunt injured solid abdominal organs [2]. The results indicate that profiling CD9^+^CD14^+^ small extracellular vesicles (EVs) collected within 24 h post-trauma could represent a novel diagnostic approach with potential clinical utility. Here, we present novel insights and addressed the new question whether monocyte-derived small EVs are sensitive enough to distinguish the severity of splenic trauma.

## Methods

### Patient collective

Seventeen adult polytrauma patients with ISS above 15 and blunt splenic injuries treated at the Level I trauma center of the German Armed Forces Central Hospital Koblenz were prospectively enrolled in this study. Twenty-eight adult polytrauma patients (ISS > 15) without splenic damage or other parenchymal organ damage (OD) were included; these patients served as negative controls (AAST 0, polytrauma w/o OD). Blood serum was collected within 24 h after polytrauma. The severity of the splenic injury was classified according to the American Association for the Surgery of Trauma Severity Grade of Splenic Injury (AAST) and the World Society of Emergency Surgery (WSES). Nonoperative management (NOM) was the treatment of choice for hemodynamic stable patients. Hemodynamically unstable patients required immediate laparotomy, either with splenectomy or packing, if they did not respond to treatment. Further details on the ‘polytrauma w/o OD’ patient cohort are available in our peer-reviewed publication in Frontiers in Immunology [2].

### Details on the methods used for small EV isolation and quantification

All detailed information on blood collection, serum isolation, small EV isolation, small EV characterization, and small EV enumeration is available in our peer-reviewed publication in Frontiers in Immunology as published in the part of the research topic ‘Community Series in Translational Insights into Mechanisms and Therapy of Organ Dysfunction in Sepsis and Trauma—Volume III’ [2].

### Ethics

The Ethics Committee of the responsible State Chambers of Medicine in Rhineland-Palatinate, Germany (ANr: 2020–15050) approved this human study. Informed consent was obtained from all the patients or their legal representatives. The presented data are part of a study that has been registered on the International Clinical Trials Registry Platform through the German Registry for Clinical Trials (DRKS 00026025; https://drks.de/search/en/trial/DRKS00026025). The current study is a new analysis focused on spleen damage. The original study was published previously in 2023 [2].

### Statistical analysis

All the data are presented as medians with 95% confidence intervals (CIs). One-way ANOVA test was used to analyze differences among the three experimental groups, followed by a post hoc test. The area under the curve (AUROC), sensitivity, specificity, and associated cutoff values were calculated. A *p* value < 0.05 was considered to indicate statistical significance. ExoView Data were processed with an ExoView® Analyzer (version 3.1.4., Unchained Labs). Statistical analysis was performed using GraphPad Prism 9 (GraphPad Software, Inc., La Jolla, USA) and the G*Power program (version 3.1.9.2., Düsseldorf, Germany). Figures were created using GraphPad Prism version 10.

## Results

**Fig. 1:**
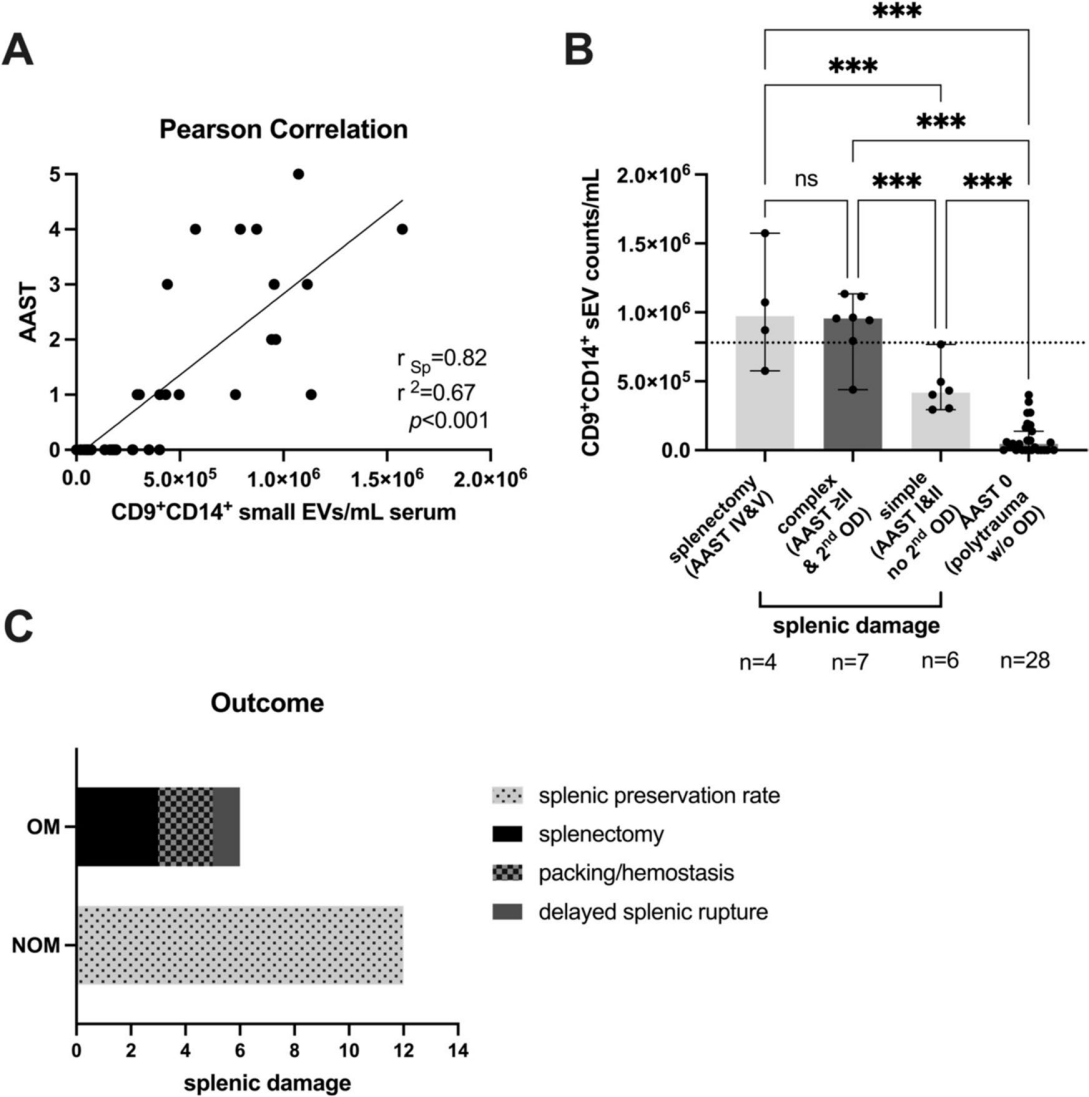
Quantification of the immunocaptured monocyte-derived CD9^+^CD14^+^ small EV subpopulation via ExoView® Tetraspanin Chips. **A** Pearson correlation between AAST score and CD9^+^CD14^+^ small EV count in polytrauma patients (ISS > 15, 24 h post-trauma) with and without splenic damage. **B** Splenic damage was subdivided into simple (AASTs I and II) and complex (AASTs ≥ II (mainly III)) with additional organ damage (OD), such as liver or kidney rupture, contusion, laceration, penetration. The splenectomy cohort underwent a splenectomy (AASTs IV and V). Polytrauma patients w/o OD (ISS > 15) served as a negative control (AAST 0). Statistical significance was assessed by simple one-way ANOVA, and multiple comparisons were performed with Fisher’s LSD test (*, *p* < 0.05; **, *p* < 0.01; ***, *p* < 0.001). Scatter dot plots showing the median and 95% CI (*n* = cohort size) are shown. The dotted line indicates the calculated cutoff between the simple and complex samples. **C** Outcome: NOM vs OM in patient outcome with splenic damage. OM—operative management was required in five cases due to hemodynamic instability and was treated with splenectomy or packing. Non-operative management (NOM) was successful in 12 cases. One patient experienced a delayed splenic rupture and subsequently underwent splenectomy

In total, 45 patients were prospectively enrolled in our study. Seventeen patients were treated for blunt splenic injury. The mean ISS among the last group was 39 ± 12.9. Among these patients, 17% (*n* = 3) had combined hepatic/splenic injury, and 23.5% (*n* = 4) had combined kidney/splenic injuries. The AAST classification was Grade I in 41.2% (*n* = 7), Grade II and III in 29.4% (*n* = 5), Grade IV in 23.5% (*n* = 4) and Grade V in 5.9% (*n* = 1). Free intraabdominal fluid was detected in 6 (35%) patients. NOM was performed in 12 (76.5%) patients, and splenectomy was performed initially in 4 (23.5%) cases. One patient had delayed spleen rupture. In this study, we showed that monocyte-derived EVs, characterized by dual positivity for CD9 and CD14 and isolated from human serum samples obtained 24 h post-trauma, exhibited a graded increase in correlation with a r_Sp_ = 0.82 (*p* < 0.001) with the severity of spleen injury; these values ranged from AAST grade 0 (no spleen damage, polytrauma w/o OD) to complex/severe spleen damage requiring operative management due to hemodynamic instability (Fig. 1A). In cases of polytrauma where there was no spleen damage (ISS > 15), predominantly involving bone fractures without additional solid organ injuries, we noted the lowest counts of CD9^+^CD14^+^ small EVs per mL of serum. Conversely, in samples from polytrauma patients with spleen lacerations classified as AAST I or II and no second solid organ injuries, such as kidney or liver injury or lung contusion, we observed significantly elevated values compared to those of patients with an AAST grade of 0, no splenic damage or any other parenchymal organ damage (OD) but largely bone fractures in polytrauma patients (ISS > 15; this patient cohort is described in detail elsewhere [2]). Incrementally elevated levels of CD9^+^CD14^+^ small EVs per mL of serum were detected in patients with complex spleen injuries, characterized as AAST grade I or higher and hemodynamic instability, often involving additional solid organ injuries. Among those with AAST IV/V, in which a splenectomy was actually performed, no significant difference was observed for complex spleen lacerations. Figure 1B shows the number of CD9^+^CD14^+^ small EVs per mL of serum, and the results were statistically significant. The detailed fold increase, AUROC, and sensitivity and specificity values are presented in Table 1. Figure 1C shows nonoperative management (NOM) vs operative management (OM) in patient outcome with splenic injury.

**Table 1:** Statistical data for CD9^+^CD14^+^ small  EVs, including the associated median, fold change, p value, sensitivity and specificity

Polytrauma w/o OD (median)	Simple (median)	Complex (median)	Splenectomy (median)	Fold	*p value	Cutoff	AUROC (*p* value)	Sensitivity [%]	Specificity [%]
46500	417160			8.97	***	283500	0.98 (< 0.001)	100	92.86
	417160	955500		2.29	***	780000	0.95 (0.007)	85.71	100
		955500	971750	1.02	n.s	959205	0.54 (0.85)	50	57.14
46500		955500		20.5	***	420425	1.0 (< 0.001)	100	100
	417160		971750	2.33	***	535500	0.96 (0.02)	100	83.33
46500			971750	20.9	***	488200	1.0 (0.001)	100	100

^*^p value: simple one-way ANOVA and multiple comparisons were performed with Fisher’s LSD test (*, *p* < 0.05; **, *p* < 0.01; ***, *p* < 0.001)

## Discussion

Our conceptual study, LiBOD (Liquid Biopsy in Organ Damage)—Spleen Laceration, offers initial insights suggesting that monocyte-derived small EVs may have the ability to distinguish between the severity grades of spleen injuries (AAST I–V) and isolated bone fractures (AAST 0) in polytrauma patients but are not specific for spleen injuries only. It is important to emphasize that these findings are based on preliminary pilot data and rather from experimental nature, thus just indicating that small EV based diagnostic may have a future in real-world usage. Nevertheless, this novel methodology needs to be validated in larger patient cohorts. In addition, yet we cannot draw any conclusions regarding future costs per samples. Nevertheless, by being the first to demonstrate the potential of monocyte-derived small EVs, even when they are present in limited quantities, and underscores the importance of further investigations. EVs have may the potential to augment extended focused assessment with sonography in trauma (E-FAST) sonography, particularly in cases of spleen laceration, by offering supplementary insights into the extent of the injury. Currently, CT and sonography (E-FAST) are commonly used modalities for diagnosing spleen lacerations [3]. CT scans are the gold standard and are generally more sensitive for detecting splenic injuries, especially when there is suspicion of significant trauma. The sensitivity and specificity of CT and sonography for diagnosing spleen lacerations can vary depending on the study and the specific criteria used. In general, CT scans have a higher sensitivity and specificity for detecting spleen injuries in adult and pediatric patients than sonography, as reviewed in the 2017 WSES classification and guidelines [1]. The sensitivity of E-FAST for detecting abdominal injuries varies from 38% to 95.4% [4]. The calculated sensitivity and specificity for CD9^+^CD14^+^ small EVs separating simple lacerations of the spleen from complex were 85.71% and 100%, respectively, in our pilot study (Table 1), supplementing our thesis. The fact that monocyte-derived extracellular vesicles (EVs) serve as an indicator of trauma severity may not be unexpected. Previous research has highlighted trauma as a complex event triggering the innate immune system and its related cascades [5]. Of particular interest is the classical inflammatory monocyte subset identified by CD14^+^CD16^−^ cells, which exhibit an elevated frequency. The mobilization of these cells correlates with the severity of the injury [6].

However, these results must be seen as a very early stage of a future exploitation. Several goals need to be taken. 1st the introduction of organ specific small EV based signature discriminating a liver injury from a spleen injury or other injuries. This might be achieved by specific small EV subpopulations, yet unknown, or by screening small EV content as for exosomal mRNA, miRNA, lncRNA, intravesicular proteins or else. Our latest publication indicated that large EVs may be not that potent as small EVs in trauma [7]. 2nd and from a technology point of view, standardization and sample processing speed must be significantly improved. The used small EV staining protocol required an overnight immobilization step of the purified small EVs on the ExoView® small EV capture chip [2]. A methodology that is good for the experimental phase but not for the final application in real world, when time is from importance except non time critical application as cancer diagnosis or projections regarding further progression.

Hence, tracking CD9^+^CD14^+^ small EVs should not replace the highly specific CT scan currently in use. Instead, this approach may enhance the effectiveness of the E-FAST, although further validation is needed. This approach could be particularly valuable in situations, where a CT scan is unavailable due to resource limitations or operational issues caused by a catastrophic event or absence of the technology. In addition, we believe that this methodology could have positive implications for both adult and pediatric patients. Although this brief report focuses on a proof-of-concept pilot study, there is a pressing need for further exploitation and validation of a specific marker for spleen-related injuries.

## Data Availability

No datasets were generated or analysed during the current study.
